# The American Society of Anesthesiologists score influences on postoperative complications and total hospital charges after laparoscopic colorectal cancer surgery

**DOI:** 10.1097/MD.0000000000010653

**Published:** 2018-05-04

**Authors:** Jae-Hyun Park, Dong-Hyun Kim, Bo-Ra Kim, Young-Wan Kim

**Affiliations:** a Department of Surgery; b Department of Internal Medicine, Yonsei University Wonju College of Medicine, Wonju, Korea.

**Keywords:** American Society of Anesthesiologists score, colonic neoplasms, complication, hospital costs, laparoscopy, rectal neoplasms

## Abstract

The aim of this study was to investigate the influence of American Society of Anesthesiologists (ASA) scores on postoperative complication rates and total hospital charges following laparoscopic surgery for colorectal cancer.

All patients (n = 664) underwent laparoscopic colorectal cancer surgery. A group of patients with an ASA score of 1 or 2 (n = 575) and a group of patients with an ASA score of 3 (n = 89) were compared.

The mean age was higher in the group of patients with an ASA score of 3 than in the group of patients with an ASA score of 1 or 2 (70 vs 67 years). The rate of ICU admission (27% vs 15%) was higher in the ASA score 3 group. The mean hospital stay (14 vs 12 days) was longer in the ASA score 3 group. Postoperative 30-day complications (38% vs 27%), 30-day mortality (2% vs 0%), and a Clavien–Dindo classification of ≥3 (21% vs 11%) occurred more frequently in the ASA score 3 group. Mean total hospital charges were significantly higher in the ASA score 3 group (13,906 vs 11,575 USD). Independent risk factors that affected postoperative complications were older age [≥80 years, hazard ratio (HR) = 2.8], an ASA score of 3 (HR = 1.6), and the presence of a primary rectal tumor (HR = 1.6). Postoperative complication rates were 21.9%, 28.5%, and 38.2% in the ASA score 1, 2, and 3 groups, respectively. Total hospital charges were 14,376 USD and 10,877 USD in the groups with and without postoperative complications, respectively. Mean total hospital charges were 10,769 USD, 11,756 USD, and 13,906 USD in the ASA score 1, 2, and 3 groups, respectively.

Preoperative ASA scores may be a predictor of postoperative complications and hospital costs when planning laparoscopic surgery for colorectal cancer.

## 1 Introduction

Preoperative risk assessments for cancer patients are essential for making optimal treatment decisions. The American Society of Anesthesiologists (ASA) scoring system is a 6-category scale and is widely used to assess patients’ general preoperative health.^[1]^ The ASA score is simple to measure and is a reliable method.^[2]^ Elective colorectal surgery is usually indicated for patients with an ASA score of 1 (healthy person), an ASA score of 2 (mild systemic disease), or an ASA score of 3 (severe systemic disease). Greater ASA scores are associated with increased postoperative morbidity, such as anastomotic leakage following colorectal surgery,^[3]^ and higher risks of anesthetic complications.^[4]^

The rising cost of health care has become a primary concern of governments, hospitals, and patients in recent years. One reason for these cost increases is an increase in chronic medical and surgical illnesses in an aging society. The occurrence of postoperative adverse events is associated with increased health care costs.^[5]^ More severe postoperative complications strongly influence increased costs due to reintervention and longer hospital stays.^[6]^

In this study, we tested the hypothesis that preoperative patient performance status based on a higher ASA score may be a predictor of increased total hospital charges following cancer surgery due to the occurrence of postoperative complications. To date, few studies examining the impact of preoperative risk classification, such as ASA scoring, on hospital costs after laparoscopic surgery for colorectal cancer have been performed.^[7]^ The objective of this study was to investigate the influence of ASA scores on postoperative complication rates and total hospital charges following laparoscopic surgery for colorectal cancer.

## 2 Materials and methods

### 2.1 Patients

This retrospective clinical cohort study was performed at a tertiary university hospital and was approved by the Institutional Review Board (YWMR-15-5-046). Informed consent was obtained from all patients before surgery, and the Strengthening the Reporting of Observational Studies in Epidemiology guidelines were utilized to ensure proper reporting of this observational study.^[8]^ Eligibility criteria included histologically confirmed colorectal cancer and elective colorectal cancer surgery between January 1, 2008, and June 30, 2014. All patients (n = 664) underwent laparoscopic colorectal resection. Patients who underwent conventional open laparotomy, an emergent operation, nonresectional or bypass surgery, transanal local excision, or multivisceral resection were excluded from the study population. The attending anesthesiologist assigned the ASA classification to each patient after considering the patient's physical condition and comorbidities. Patients with ASA scores of 4 (severe systemic disease that is a constant threat to life), 5 (a moribund person not expected to survive without surgery), and 6 (declared brain-dead) were excluded from analysis. Outcomes were compared between patients with an ASA score of 1 or 2 and patients with an ASA score of 3.

### 2.2 Study objectives

The primary objective of this study was to investigate the influence of ASA scores on postoperative complication rates and total hospital charges during the index surgery. Thus, we compared postoperative complication rates and total hospital charges between patients with an ASA score of 3 and patients with an ASA score of 1 or 2 following laparoscopic surgery for colorectal cancer.

### 2.3 Preoperative chemoradiation therapy, surgery, and adjuvant therapy

Preoperative chemoradiation therapy was performed for patients with rectal cancer who were diagnosed with clinical stage T3 or T4 and/or node-positive disease.^[9]^ The choice of laparoscopic surgery was determined by an informed consultation between the surgeon and patient. All surgeries were performed by 2 experienced colorectal surgeons.^[10,11]^ After a standardized preoperative preparation, a complete mesocolic excision and central vascular ligation were performed for patients with colon cancer.^[12]^ In patients with rectal cancer, a high ligation of the inferior mesenteric artery and total mesorectal excision were performed.^[13]^ After the pathology report was completed, all patients with stage II, III, and IV disease were advised to undergo chemotherapy according to the National Comprehensive Cancer Network (NCCN) guidelines.^[14,15]^

### 2.4 Outcome variables

All data were analyzed according to the intention-to-treat principle. Postoperative surgical complications were defined as the events that required additional treatment within 30 days after surgery. The severity of surgical complications was evaluated based on the Clavien–Dindo classification (grades I to V).^[16]^ On the basis of the Dindo-Clavien classification, a grade II complication was defined as a condition requiring pharmacological treatment. A grade III complication was defined as a condition requiring surgical, endoscopic, or radiological intervention. According to the Centers for Disease Control and Prevention guidelines, surgical site infection (SSI) was classified as superficial incisional, deep incision, or organ/space SSI.^[17]^ All postoperative complications were recorded within 30 days following the surgery. The length of hospital stay was defined as the number of nights from the date of surgery to discharge. Outcome variables, such as intensive care unit (ICU) care or blood transfusions, were included if they were required within 48 hours after the index surgery. Binary cutoffs for continuous variables, such as a lengthy operative time (≥300 minutes)^[18]^ and a greater amount of operative blood loss (≥150 mL),^[19]^ were decided on the basis of previously published studies.

Health care cost evaluations categorize costs as direct, indirect, or intangible. Direct costs are directly attributable to an intervention for diseases. Indirect costs are productivity losses caused by disease-related morbidity and premature mortality. Intangible costs refer to pain, suffering, grief, and other nonfinancial outcomes associated with illness.^[20]^ We calculated the direct costs of laparoscopic surgery for colorectal cancer from the patient perspective based on the billed costs. Total hospital charges included overall charges requested during hospitalization for the index cancer surgery. Operation charges included charges for the operation, anesthesia, intraoperative medications, laboratory, and radiology tests. Procedure charges included charges for postoperative dressing and intravenous-line care. Total hospital charges, operation charges, and procedure charges were expressed in US Dollars (USDs) at the April 2017 exchange rate (1144 Korean Won = 1 USD).

### 2.5 Statistical analysis

All statistical analyses were performed with IBM SPSS Statistics for Windows, version 21.0 (IBM Corporation, Armonk, NY) and MedCalc Statistical Software version 17.4 (MedCalc Software bvba, Ostend, Belgium; http://www.medcalc.org; 2017). Categorical variables were described by frequencies and percentages and compared using a Chi-square or Fisher exact test as appropriate. Continuous variables are described as the mean and standard deviation and were analyzed by Student *t* tests and analyses of variance (ANOVAs). Factors associated with 30-day complications were assessed by logistic regression analysis. A *P* value of <.05 was considered significant.

Univariate logistic regression analyses were performed with all variables, and variables with a *P* value of <.05 were used for multivariable analyses. Multivariable logistic regression analyses were performed by the forward stepwise selection of variables.

## 3 Results

### 3.1 Patient characteristics

Among 664 patients, 105, 470, and 89 patients were classified as ASA score 1, 2, and 3, respectively. Patients with an ASA score of 1 or 2 (n = 575) were compared with patients with an ASA score of 3 (n = 89). The mean age was higher in the ASA score 3 group (70 ± 10 years) than in the ASA score 1 and 2 group (67 ± 11 years, *P* = .02).

The rate of ICU admission (27% vs 15%, *P* = .003) was higher in the ASA score 3 group. The mean hospital stay (14 ± 8 vs 12 ± 6, *P* = .016) and mean time to soft diet (7 ± 4 vs 5 ± 3 days, *P* = .046) were significantly longer in the ASA score 3 group. Postoperative 30-day complications (38% vs 27%, *P* = .035), 30-day mortality (2% vs 0%, *P* = .007), and a Clavien–Dindo classification of ≥3 (21% vs 11%, *P* = .007) were more frequent in the ASA score 3 group. Mean total hospital charges were significantly higher in the ASA score 3 group (13,906 vs 11,575 USD, *P* = .039). Detailed patient characteristics are summarized in Table 1.

**Table 1:**
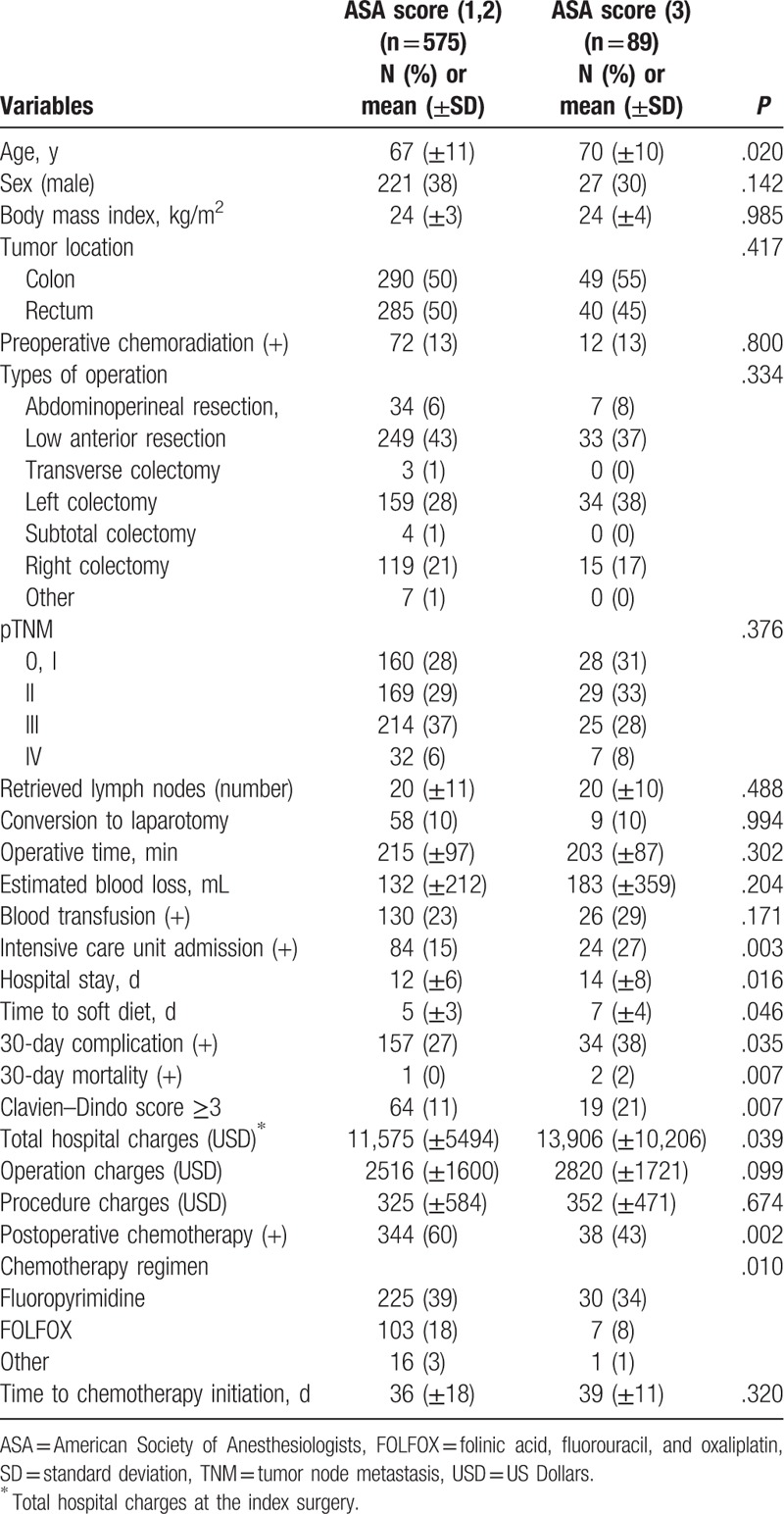
Patient characteristics (n = 664).

### 3.2 Preoperative comorbidities

The most common comorbidity was cardiovascular disease. The ASA score 3 group showed higher rates of diabetes (38% vs 20%, *P* < .001), cardiovascular disease (75% vs 46%, *P* < .001), and kidney disease (4% vs 1%, *P* = .001) than the ASA score 2 group. The frequency of 2 or more comorbidities was higher in the ASA score 3 group (64% vs 20%, *P* < .001) (Table 2).

**Table 2:**
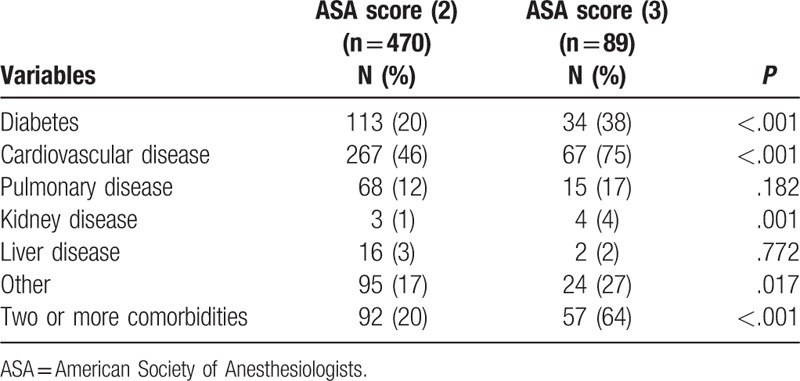
Preoperative comorbidities.

### 3.3 Postoperative complications

The overall postoperative complication rate was higher in the ASA score 3 group; however, there were no significant differences regarding specific complications between the 2 groups in terms of cardiovascular (*P* = .441), pulmonary (*P* = .063), or gastrointestinal (*P* = .377) complications. The SSI rate was nonsignificantly higher in the ASA score 3 group (15.7% vs 11.8%, *P* = .182) (Table 3).

**Table 3:**
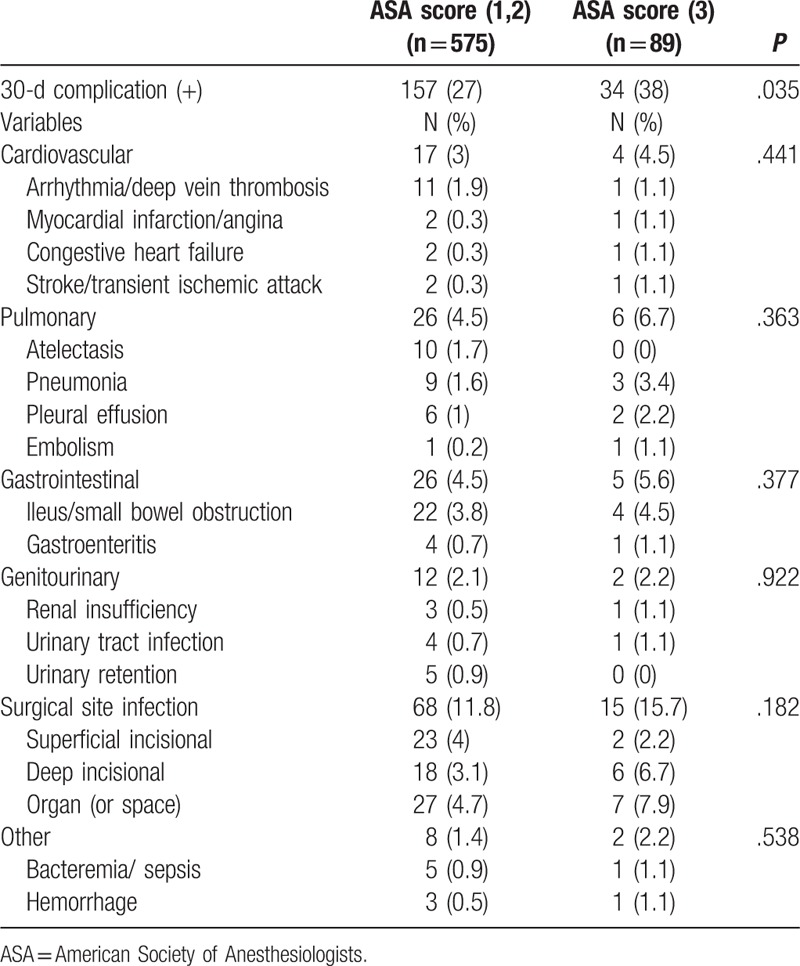
Type of postoperative complications.

### 3.4 Predictors for postoperative complications

On the basis of the multivariable logistic regression analysis, the independent risk factors that affected postoperative complications were older age [≥80 years, hazard ratio (HR) with 95% confidence interval (95% CI) = 2.8 (1.2–6.6), *P* = .020], an ASA score of 3 [HR with 95% CI = 1.6 (1.1–2.4), *P* = .021], and the presence of a primary rectal tumor [HR with 95% CI = 1.6 (1.1–2.4), *P* = .008] (Table 4).

**Table 4:**
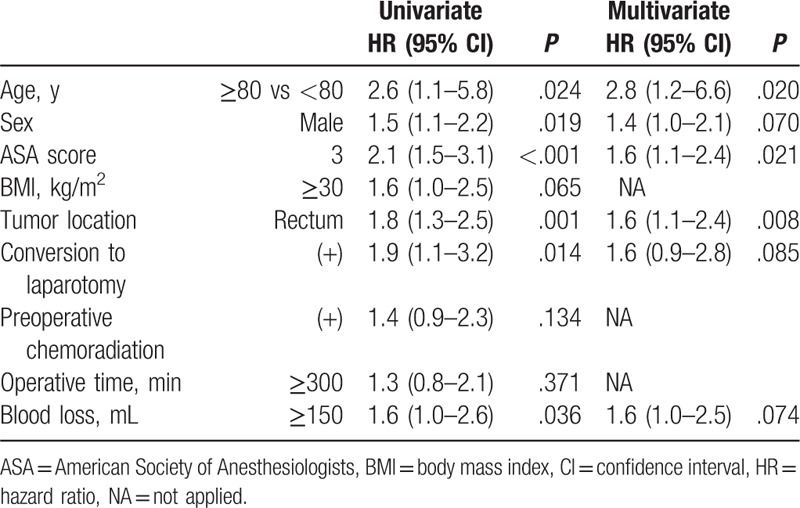
Predictors for postoperative complication using logistic regression analysis.

### 3.5 Association between ASA score and postoperative complication rate

**Figure 1:**
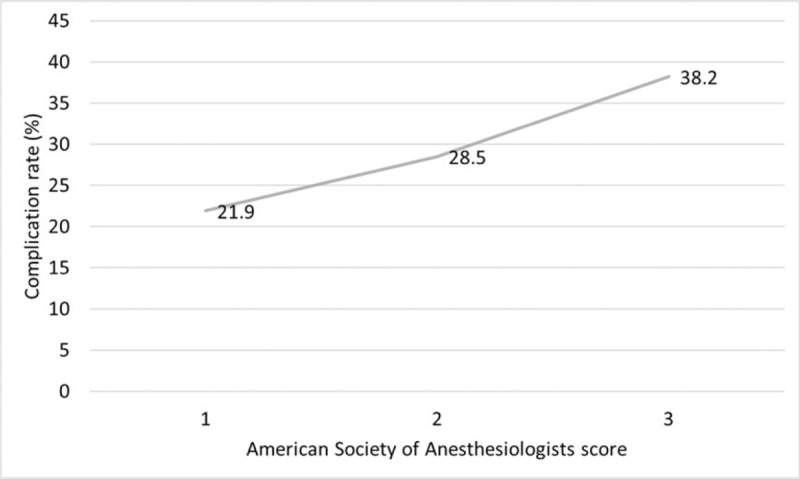
Association between American Society of Anesthesiologists scores and postoperative complication rates. Rates of postoperative complications gradually increased as the American Society of Anesthesiologists scores increased (*P* = .035).

Rates of postoperative complications gradually increased according to the ASA score (*P* = .035). The postoperative complication rate was 21.9%, 28.5%, and 38.2% in the ASA score 1, 2, and 3 groups, respectively (Fig. 1).

### 3.6 Association between the occurrence of postoperative complications and total hospital charges during the index surgery

**Figure 2:**
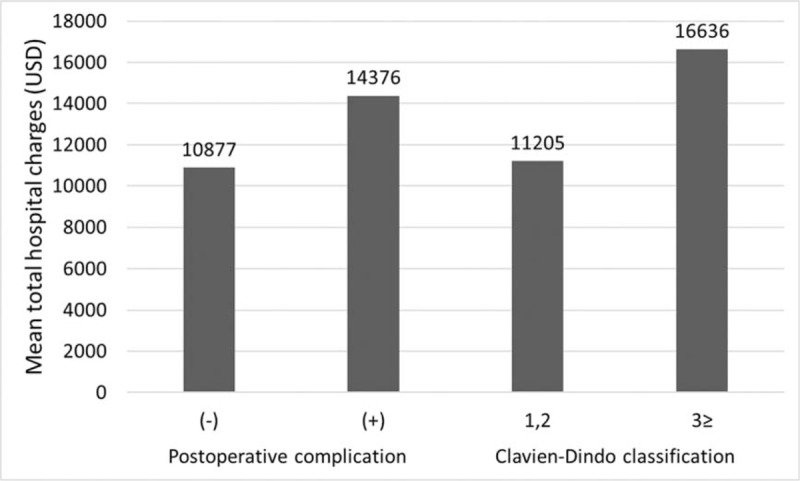
Association between the occurrence of postoperative complications and total hospital charges during the index surgery. Increased mean total hospital charges are associated with the occurrence of postoperative complications (*P* < .001) and more severe complications (Clavien–Dindo score ≥3, *P* < .001).

Increased mean total hospital charges are associated with the occurrence of postoperative complications (*P* < .001) and more severe complications (Clavien–Dindo score ≥3, *P* < .001). Total hospital charges were 14,376 USD and 10,877 USD among patients with and without postoperative complications, respectively (Fig. 2).

### 3.7 Association between the ASA score and total hospital charges during the index surgery

**Figure 3:**
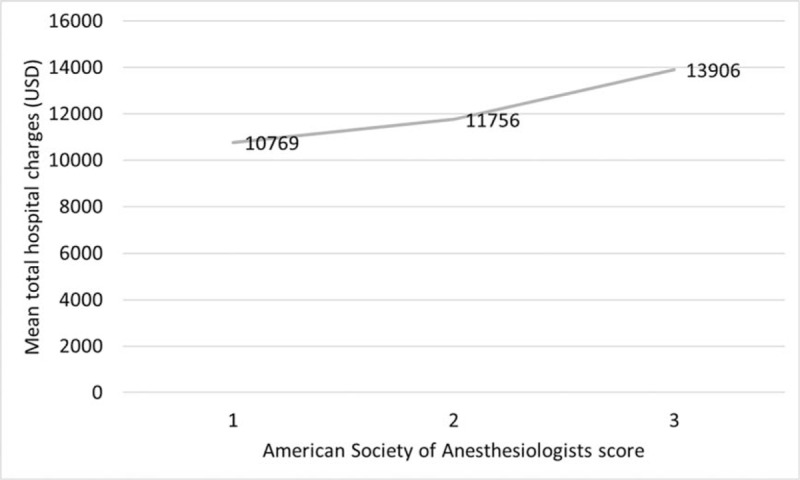
Association between the American Society of Anesthesiologists scores and total hospital charges during the index surgery. A higher American Society of Anesthesiologists score is associated with higher mean total hospital charges (*P* = .002).

A higher ASA score is associated with an increased amount of mean total hospital charges (*P* = .002). Mean total hospital charges were 10,769 USD, 11,756 USD, and 13,906 USD in the ASA score 1, 2, and 3 groups, respectively (Fig. 3).

## 4 Discussion

The major findings of this study are that postoperative complication rates significantly increase with the ASA score following laparoscopic surgery for colorectal cancer. Increased mean total hospital charges are associated with the occurrence of postoperative complications and more severe complications (Clavien–Dindo score ≥3). In addition, a higher ASA score is associated with increased mean total hospital charges. Thus, the preoperative ASA score may be used as a predictor for postoperative complications and hospital costs when planning laparoscopic surgery for colorectal cancer. Colorectal surgery for patients at high risk will increase as time goes by, and ASA scoring is potentially a practical method for the estimation of health care costs. Preoperative high-risk conditions are usually difficult to modify, but patients at high risk may be preoperatively informed about potential morbidity and high cost when planning cancer surgery.

First, we evaluated the association between ASA scores and postoperative complication rates and found that the rates of postoperative complications gradually increased according to the ASA scores. The ASA score is a convenient method for the assessment of performance status because it summarizes multiple patient characteristics.^[21]^ A patient's physical condition and age are closely related. The incidence of colorectal cancer increases with age, reaching a peak at approximately 80 years of age.^[22]^ The chronological aging process diminishes the homeostatic reserve of diverse organ systems and results in increased susceptibility to chronic illnesses in elderly patients.^[23]^ Indeed, patients in the ASA score 3 group were older and had higher incidences of diabetes, cardiovascular disease, kidney disease, and 2 or more comorbidities in this study. Previous studies indicated that a higher ASA score was a risk factor for SSI and anastomotic leakage.^[24]^ We also found that an ASA score of 3 was an independent risk factor for postoperative complications. Therefore, benefits and risks of surgical treatment should be communicated preoperatively to this fragile group of patients due to their frailty caused by reduced physiological function and frequent comorbidities.^[25,26]^

Second, we evaluated the association between the occurrence of postoperative complications and total hospital charges during the index surgery and found that increased mean total hospital charges were associated with the occurrence of postoperative complications and more severe complications. In the absence of surgical complications or when complications are mild, a shorter hospital stay and a lower need for reintervention can reduce hospital costs.^[5]^

Finally, we evaluated the association between ASA scores and total hospital charges during the index surgery and found that a higher ASA score was associated with an increased amount of mean total hospital charges. Surgery for high-risk patients is expected to be associated with increased costs due to the negative effects of associated comorbidities on complicated recovery, morbidity, and length of hospital stay. Govaert et al^[7]^ compared actual 90-day hospital costs between elective open and laparoscopic colon and rectal cancer resection. In their subgroup analysis, patients with ASA scores of 3 or 4 had lengthier hospital stays than patients with ASA scores of 1 or 2 (8.4 vs 10.3 days in colon cancer, 11.5 vs 16.1 days in rectal cancer). Postoperative complication rates were higher in the ASA 3 and 4 group (21.4% vs 13.3% in colon cancer, 37.7% vs 20.9% in rectal cancer). Finally, total hospital costs in the ASA 3 and 4 group were higher than in the ASA 1 and 2 group. Similarly, we observed that the ASA score 3 group showed higher rates of ICU admission within 48 hours, due to either preoperative planned stay or intraoperative decision, and experienced a prolonged hospital stay. These factors might have contributed to increased hospital charges.

This study has several limitations. This study contains a small sample size from a single institution. The documentation of postoperative complications may be biased due to the retrospective nature of this study. This analysis is also limited by our choice of cost methodology, as only direct costs were included from single hospital data. In addition, health care costs and insurance systems are quite different among countries. These factors might diminish the statistical power of the study and compromise the generalizability of the current findings. However, this is the second effort to correlate the ASA score with both the occurrence of postoperative complications and hospital costs after laparoscopic surgery for colorectal cancer.^[7]^

In summary, this study revealed that patients’ ASA scores influenced postoperative complication rates and total hospital charges following laparoscopic surgery for colorectal cancer. On the basis of our results, patients with high-risk conditions may be informed preoperatively of their potential higher morbidity and cost when planning cancer surgery. Future studies with larger cohorts would be helpful to confirm the current findings of this study.

## Acknowledgment

The authors thank Ik Yong Kim for performing surgery and his surgical cases are included in database and Hyun Jun Kwon for the management of the colorectal database, without which this study would not have been possible. This research was supported by Basic Science Research Program through the National Research Foundation of Korea (NRF) funded by the Ministry of Education (NRF-2017R1D1A3B03032301).

## Author contributions

**Conceptualization:** Jae-Hyun Park, Dong-Hyun Kim, Bo-Ra Kim, Young-Wan Kim.

**Data curation:** Jae-Hyun Park, Dong-Hyun Kim, Bo-Ra Kim, Young-Wan Kim.

**Formal analysis:** Jae-Hyun Park, Dong-Hyun Kim, Bo-Ra Kim, Young-Wan Kim.

**Investigation:** Jae-Hyun Park, Bo-Ra Kim, Young-Wan Kim.

**Methodology:** Jae-Hyun Park, Dong-Hyun Kim, Bo-Ra Kim, Young-Wan Kim.

**Validation:** Dong-Hyun Kim, Bo-Ra Kim, Young-Wan Kim.

**Visualization:** Bo-Ra Kim, Young-Wan Kim.

**Writing – original draft:** Jae-Hyun Park.

**Writing – review & editing:** Dong-Hyun Kim, Bo-Ra Kim, Young-Wan Kim.

Substantial contributions to conception and design, acquisition of data, or analysis and interpretation of data; PJH, KBR, KDH, KYW; Drafting the article or revising it critically for important intellectual content; PJH, KBR, KDH, KYW; Final approval of the version to be published; PJH, KBR, KDH, KYW; Agreement to be accountable for all aspects of the work in ensuring that questions related to the accuracy or integrity of any part of the work are appropriately investigated and resolved; PJH, KBR, KDH, KYW
